# Supplementary material for: Relationship between visual function and cognitive function in the elderly: A cross-sectional observational study
Source: PLoS One. 2020 May 19;15(5):e0233381. doi: 10.1371/journal.pone.0233381 (PMC7236991; doi:10.1371/journal.pone.0233381)
Supplement: Attachment — Submitted filename: 200328Response PONE-D-20-030980.docx [file pone.0233381.s002.docx]

Reviewer #1: The topic of this study is refreshing to focus on the relationship between visual ability and cognitive function. The results reported are still somewhat exploratory and needed to be examined further due to the following aspects:

1) The allocation of subjects is a bit confusing. In Cognitive Function, “a score<=23 indicates a possibility of dementia…, and a score of 24-28 indicates a possibility of MCI”. However, in discussion section, subjects with a MMSE sore<28 were allocated to a MCI group. How did the author consider here? Or was it not rigorous enough.

Thank you for pointing it out. It was just an unintended mistake. Thus, we revised it as follows: “…a score of 24–27 indicates a possibility of mild cognitive impairment...”

2) When the first paragraph of “Discussion” was read, this reviewer understood that the higher-order aberration was used to exclude the optical factor. It would be clearer that the author explained previously in “Measurement of wavefront aberrations”. It is regrettable that this reviewer still do not understand the role of Schirmer value.

Please note that we explained the purpose for the inspection of wavefront aberrations as follows: “Higher-order aberrations were measured using the Hartmann-Shack wavefront aberrometer (Topcon Corp., Tokyo, Japan) to evaluate each subject’s optical quality.”

We also added the explanation of Schirmer test as follows: “The Schirmer test was performed without topical anesthesia using a sterilized Schirmer strip (Whatman No. 41, Ayumi Pharmaceutical Corporation, Tokyo, Japan) after completion of all other examinations to confirm the presence or absence of dry eye, which may affect visual function.”

3) The author focused on the measurement of cognitive function and visual ability. But there are many other external factors affecting them, such as driving performance, road environment, traffic condition...More researches are needed. The finding was expected to be used to reduce road traffic rate and assess visual performance in elderly drivers. Most analyses focused on explaining the relationship from a medical perspective, not from a perspective of accident prevention. And 34 subjected selected were unclear whether they were drivers.

We agree with you. Thus, we added more details in the limitation section as follows: “First, the number of subjects included was small. Moreover, the subjects were not limited to drivers. Second, we focused solely on measurement of cognitive function and visual ability and did not assess the relationship between actual driving performance and cognitive and visual function. However, many other external factors influence the risk of an RTA, such as driving performance [41,42], environmental road conditions [43,44], and traffic conditions [45,46].”

4）Detailed the experiment section, such as providing the necessary figures to show the experiment and equipment, explaining the experiment procedure.

Please note that Fig 1 is provided to show (A) Equipment (AS-28) used to measure functional visual acuity. (B) Optotype display on the functional visual acuity system. (C) The results presented in graphical form.

We also presented the representative cases with MCI and NC in Figure 4.

5) Statistical analysis used in the article was simple. The results shown in figure 2, R or R2？Anyhow, all the values are not good enough, maybe which is due to the small data samples. Please provide more explanation or comparison to previous studies. The figures need to be redrawn, such as adding axis lables.

Thank you for your constructive comment. The study sample is small for the statistical analysis. Thus, in the analysis of 2-group comparison, we confirmed that data is equally distributed using F-test, and then conducted the analysis of 2-group comparison by using t-test. In the correlation of MMSE scores and functional VA parameters, we conducted a test of no correlation. Correlation coefficient, test statistics and P values were -0.362, 2.195 and 0.036 between MMSE scores and functional VAs, 0.340, 2.052 and 0.0485 between MMSE scores and VMRs, -0.346, 2.089 and 0.0448 between MMSE scores and SD of functional VAs, respectively. Hence, the null hypothesis was rejected, and the data was not uncorrelated.

Please note that we added the additional statistical methods in the statistical section as follows: “The functional VA parameters, total higher-order aberrations, and Schirmer values were compared between the MCI and NC groups. The Student’s *t*-test was used for between-group comparisons after confirming that the data were equally distributed using the F-test. The relationship between the functional VA parameters and MMSE scores in the study population overall was examined by Pearson’s correlation analysis. A further test of the significance of the correlation coefficient was performed to confirm whether or not the null hypothesis would be rejected.” With the additional information, we added in the result section of “Correlation between cognitive function and visual function” as follows: “Further tests of significance for the correlation coefficient, test statistics, and P-values were 2.195 and 0.036 for MMSE scores and functional VAs, 2.052 and 0.0485 for MMSE scores and the visual maintenance ratio, and 2.089 and 0.0448 for MMSE scores and the SD of functional VAs, respectively, demonstrating that the null hypothesis was rejected.”

We added the new figure of the distribution of the functional VA parameters.

We also cited more references to show the relationship between cognitive and visual functions in the visually asymptomatic subjects as follows: “There have been reports of subclinical visual impairment in visually asymptomatic patients using high sensitivity methods such as contrast sensitivity testing [37-39] that are consistent with our findings. Wieder et al. showed that cognitive function not only correlated with low contrast sensitivity but also with information processing speed and memory among the cognitive domains affecting visual performance [39].”

We added the axis labels in Fig. 5.

6) The conclusion section is missing, maybe the current analysis section could be divided into two sections.

Please note that we mentioned in the section of statistical analysis that each function of the MMSE test was compared between the two groups. We also divided into two sections as follows: “Subjects with an MMSE score <28 were allocated to an MCI group and those with an MMSE score ≥28 to a normal cognition (NC) group. Characteristics of cognitive functions, such as orientation to time and place, registration, attention and calculation, recall, language, and copying, were compared between the two groups.

The functional VA parameters, total higher-order aberrations, and Schirmer values were compared between the MCI and NC groups. The Student’s *t*-test was used for between-group comparisons after confirming that the data were equally distributed using the F-test. The relationship between the functional VA parameters and MMSE scores in the study population overall was examined by Pearson’s correlation analysis. A further test of the significance of the correlation coefficient was performed to confirm whether or not the null hypothesis would be rejected.”

Reviewer #2: The topic of this paper is interesting and the methods sound. The results are useful and meaningful. There are several suggestions to improve this paper.

1. Some references are needed for student’s test. And the authors need to mention that why student’s test is used instead of ANOVA. The authors could refer to the following ones.
[1] Examining the safety of trucks under crosswind at bridge-tunnel section: A driving simulator study, Tunnelling and Underground Space Technology, 2019, 92, 103034. https://doi.org/10.1016/j.tust.2019.103034
[2] Examining the influence of decorated sidewaall in road tunnels using fMRI technology, Tunnelling and Underground Space Technology, Volume 99, 2020, <https://doi.org/10.1016/j.tust.2020.103362>

Please note that we cited the references, showing the studies of 2-group comparison using t-test as follows: “There have been reports of subclinical visual impairment in visually asymptomatic patients using high sensitivity methods such as contrast sensitivity testing [37-39] that are consistent with our findings. Wieder et al. showed that cognitive function not only correlated with low contrast sensitivity but also with information processing speed and memory among the cognitive domains affecting visual performance [39].”

We also changed the statistical methods and procedures clearly in the section of Statistical analysis as follows: “Subjects with an MMSE score <28 were allocated to an MCI group and those with an MMSE score ≥28 to a normal cognition (NC) group. Characteristics of cognitive functions, such as orientation to time and place, registration, attention and calculation, recall, language, and copying, were compared between the two groups.

The functional VA parameters, total higher-order aberrations, and Schirmer values were compared between the MCI and NC groups. The Student’s *t*-test was used for between-group comparisons after confirming that the data were equally distributed using the F-test. The relationship between the functional VA parameters and MMSE scores in the study population overall was examined by Pearson’s correlation analysis. A further test of the significance of the correlation coefficient was performed to confirm whether or not the null hypothesis would be rejected.”

We wrote the limitation more clearly as follows: “Our study has a few limitations. First, the number of subjects included was small. Moreover, the subjects were not limited to drivers. Second, we focused solely on measurement of cognitive function and visual ability and did not assess the relationship between actual driving performance and cognitive and visual function. However, many other external factors influence the risk of an RTA, such as driving performance [41,42], environmental road conditions [43,44], and traffic conditions [45,46]. Therefore, more research is needed in a large number of subjects to investigate the relationship between MCI and risk of an RTA.”

We also cited the articles as you recommended.

2. For Fig.1, Box-plot with outlier maybe is preferred.

 Please note that the figure was changed to box-plot with outlier, as recommended.
